# Lethality of complex neuronal network in *Caenorhabditis elegans* nervous system based on cell attacks

**DOI:** 10.1186/1471-2202-13-S1-P110

**Published:** 2012-07-16

**Authors:** Seongkyun Kim, Hyoungkyu Kim, Jaeseung Jeong

**Affiliations:** 1 Department of Bio and Brain Engineering, Korea Advanced Institute of Science and Technology (KAIST), Daejeon 305-701, South Korea

## 

**Figure 1:**
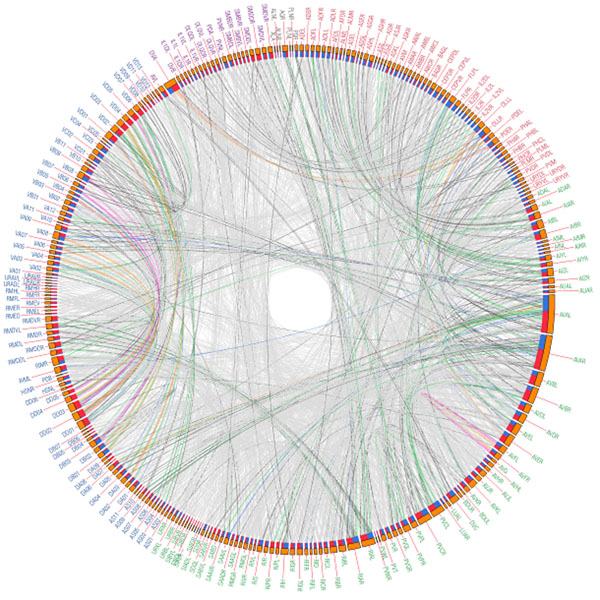
Directed circular wiring diagram of the *C. elegans* combined network. The colors of the links showed the weights of each connection (Color: Weight ranges; light grey: 1-5, grey: 6-10, green: 11-15, blue: 16-20, orange: 21-25, pink: 26-30, and red: over 30). The color of the name of neuron indicated their neuronal type [1,2] (sensory: red, inter: green, motor: blue, polymodal: purple, and unknown: grey). The links departed from blue segment of a neuron and arrived to red segment of a neuron. The lengths of the orange segments indicated total synaptic strengths (weights) of neurons.

The aim of this study was to investigate changes in network structural properties and functional perturbations of the *C. elegans* network which were induced by simulated lesions of the neural network through removals of each single neuron (attacks). We analyzed complete neuronal wiring data (i.e. connectome) of the nematode *C. elegans *[1] consisting of 279 neurons (nodes) and their connections (edges). We constructed the circular wiring diagram of simply combined network of gap junctions and chemical synapses as shown Figure 1. Then, we measured several measures of complex network properties of directed weighted neuronal network of *C. elegans* to examine the effect of single node attack: the clustering coefficient, global efficiency, isolated nodes, and reachability [2]. We found that the deletions of motor neurons and interneurons were more effective to the clustering coefficient of the network than the sensory neurons. Eliminations of some interneurons mainly decreased global efficiency, and remarkably increased global efficiencies were induced by each removal of sensory neurons (see Table 1). We suggest that this complex network analysis of the c. elegans connectome is helpful for understanding the potential functions of all neurons, and provide insight into which neurons are crucial for specific functions and which neurons are critical for lethality of the network information processing.

**Table 1:** The average clustering coefficient and the average global efficiency when a target node deleted. A deletion of one of listed neurons induced remarkable changes in each measure.

**Clustering Coefficient (CC)**						**Global Efficiency (GE)**				
		
Average of CC in original net: 0.643						Average of GE in original net: 1.055				
		
**Increased (176)^+^**			**Decreased (103)**			**Increased (134)**			**Decreased (145)**	
						
DVB(M) ^++^	0.655		AVAR(I)	0.548		IL2DR(S)	1.060		AVAL(I)	0.991
VD10(M)	0.653		AVAL(I)	0.558		IL2DL(S)	1.060		AVAR(I)	1.005
RID(M)	0.651		AS08(M)	0.624		PLNR(S)	1.059		DVA(I)	1.030
HSNR(M)	0.650		VA08(M)	0.631		URADR(S)	1.059		PVCL(I)	1.031
AVL(M)	0.650		VB08(M)	0.631		URAVR(S)	1.059		PVCR(I)	1.033

^+^ The number of the neurons who have increased values of each measurement than the value of original network

^++^ S: Sensory neuron, I: Interneuron, M: Motor neuron
